# The Reparative Abilities of Menstrual Stem Cells Modulate the Wound Matrix Signals and Improve Cutaneous Regeneration

**DOI:** 10.3389/fphys.2018.00464

**Published:** 2018-05-14

**Authors:** Jimena Cuenca, Alice Le-Gatt, Valentina Castillo, Jose Belletti, Macarena Díaz, Mónica Kurte G, Paz L. Gonzalez, Francisca Alcayaga-Miranda, Christina M. A. P. Schuh, Fernando Ezquer, Marcelo Ezquer, Maroun Khoury

**Affiliations:** ^1^Consorcio Regenero, Chilean Consortium for Regenerative Medicine, Santiago, Chile; ^2^Laboratory of Nano-Regenerative Medicine, Faculty of Medicine, Universidad de los Andes, Santiago, Chile; ^3^Cells for Cells, Santiago, Chile; ^4^Laboratory of Pathological Anatomy, Hospital DIPRECA, Las Condes, Chile; ^5^Laboratory of Immunology, Faculty of Medicine, Universidad de los Andes, Santiago, Chile; ^6^Centro de Medicina Regenerativa, Facultad de Medicina Clínica Alemana-Universidad del Desarrollo, Santiago, Chile

**Keywords:** menstrual stem cells, mesenchymal stem cells, wound healing, angiogenesis, regeneration, cell therapy

## Abstract

Considerable advances have been made toward understanding the cellular and molecular mechanism of wound healing, however, treatments for chronic wounds remain elusive. Emerging concepts utilizing mesenchymal stem cells (MSCs) from umbilical cord, adipose tissue and bone marrow have shown therapeutical advantages for wound healing. Based on this positive outcome, efforts to determine the optimal sources for MSCs are required in order to improve their migratory, angiogenic, immunomodulatory, and reparative abilities. An alternative source suitable for repetitive, non-invasive collection of MSCs is from the menstrual fluid (MenSCs), displaying a major practical advantage over other sources. This study aims to compare the biological functions and the transcriptomic pattern of MenSCs with umbilical cord MSCs in conditions resembling the wound microenvironment. Consequently, we correlate the specific gene expression signature from MenSCs with changes of the wound matrix signals *in vivo*. The direct comparison revealed a superior clonogenic and migratory potential of MenSCs as well as a beneficial effect of their secretome on human dermal fibroblast migration *in vitro*. Furthermore, MenSCs showed increased immunomodulatory properties, inhibiting T-cell proliferation in co-culture. We further, investigated the expression of selected genes involved in wound repair (growth factors, cytokines, chemokines, AMPs, MMPs) and found considerably higher expression levels in MenSCs (ANGPT1 1.5-fold; PDGFA 1.8-fold; PDGFB 791-fold; MMP3 21.6-fold; ELN 13.4-fold; and MMP10 9.2-fold). This difference became more pronounced under a pro-inflammatory stimulation, resembling wound bed conditions. Locally applied in a murine excisional wound splinting model, MenSCs showed a significantly improved wound closure after 14 days, as well as enhanced neovascularization, compared to the untreated group. Interestingly, analysis of excised wound tissue revealed a significantly higher expression of VEGF (1.42-fold) among other factors, translating an important conversion of the matrix signals in the wound site. Furthermore, histological analysis of the wound tissue from MenSCs-treated group displayed a more mature robust vascular network and a genuinely higher collagen content confirming the pro-angiogenic and reparative effect of MenSCs treatment. In conclusion, the superior clonogenicity, immunosuppressive and migration potential in combination with specific paracrine signature of MenSCs, resulted in an enhanced wound healing and cutaneous regeneration process.

## Introduction

Although considerable advances have been made concerning understanding the process of wound healing, as well as cellular and molecular responses involved, treatment of chronic wounds remains elusive. Especially, patients with comorbidities such as diabetes are prone to face complications within the triad of ischemia, neuropathy, and infection (Eldor et al., 2004). This results in frequent and prolonged hospitalization and displays a major burden to patients and the social health-care systems. Traditional approaches to initiate regeneration include sterile wound dressings, repeated debridement of necrotic material and administration of antibiotics to decrease the bacterial load (Werdin et al., 2008). Among the various factors contributing to non-healing wounds play a key factor the reduced angiogenesis and the dysregulation in the production of cytokines by local immune cells and fibroblast (Otero-Viñas and Falanga, 2016).

Over the last decades, novel approaches using therapeutic cells have become increasingly popular and proven to be promising in tissue regeneration. In particular, mesenchymal stem cells (MSCs) have been shown to express a number of beneficial characteristics for wound healing. MSCs are tissue resident progenitor cells with self-renewable, immune-modulatory, anti-inflammatory characteristics as well as the capability of differentiating into a number of cell types. Sources for MSCs are abundant in the human bodies, as they can be found in virtually every tissue (bone marrow, adipose tissue, dental pulp, umbilical cord, amniotic membrane…). However, increasing evidence suggests that MSCs are prone to explant site-dependent differences, ranging from secretion of growth factors, differentiation potential, immunosuppressive properties, migration or expression of specific cell surface markers (Hass et al., 2011; Nombela-Arrieta et al., 2011; Al-Nbaheen et al., 2013; Wegmeyer et al., 2013). Depending on the intended application, these differences can be utilized to maximize the effect of transplanted cells.

The gold standard in clinical trials so far have been bone marrow derived MSCs (BM-MSCs), but also adipose derived MSCs (ADSCs), as well as umbilical cord derived MSCs (UC-MSCs) (Squillaro et al., 2016; Wang et al., 2016). All of these sources, however, show distinct disadvantages as they are obtained by either painful and invasive procedures or single donations.

Based on the positive outcome of the use of MSCs in wound healing, efforts to determine the optimal sources for MSCs are still needed to improve their migratory, angiogenic, immunomodulatory, and reparative abilities. An alternative source for repetitive, non-invasive collection and without ethical concerns are menstrual blood-derived mesenchymal stem cells (MenSCs) (Meng et al., 2007). These characteristics are potentially what makes MenSCs an attractive MSCs source for cell therapy. Aside from the classic MSCs traits, MenSCs compared with BM-MSCs exhibit characteristics such as increased migration and angiogenesis making them favorable for application in wound healing (Alcayaga-Miranda et al., 2015a). Our group and others have described the therapeutic potential of allogenic MenSCs derived from healthy donors in different preclinical studies such as sepsis (Alcayaga-Miranda et al., 2015b), Graft-Vs.-Host-Disease (Luz-Crawford et al., 2016b), acute lung injury (Xiang et al., 2017), ischemic stroke (Rodrigues et al., 2012), Duchenne muscular dystrophy (Cui et al., 2007) or liver fibrosis (Chen et al., 2017), supporting the promising use for clinical applications. While the capacity of MenSCs to differentiate *in vitro* to epidermal lineage and keratinocytes has been described (Faramarzi et al., 2016; Akhavan-Tavakoli et al., 2017), their contribution *in vivo* to wound healing treatment remains unclear. Besides, UC-MSCs have been shown to exhibit enhanced therapeutic abilities in terms of angiogenesis and cell migration when compared to BM-MSCs, suggesting that UC-MSCs might be a better source of MSCs for tissue repair (Hsieh et al., 2013). Therefore, this study aims to compare first the biological functions and the specific transcriptomic pattern of different secreted factors from MenSCs with UC-MSCs, in conditions resembling the wound microenvironment. Consequently, we correlate the specific gene expression signature from MenSCs with the changes occurred in the wound healing milieu *in vivo*.

## Materials and Methods

### Ethics Statement

This work was revised and approved by the Ethics Committee of the Universidad de los Andes. Menstrual fluids and umbilical cord were collected from healthy donors after written informed consent following institutional guidelines (Alcayaga-Miranda et al., 2015a; González et al., 2015). All animal studies were performed at the Cells for Cells Animal Facility in accordance with protocols revised and approved by the Institutional Animal Care and Use Committee of Universidad de los Andes.

### Cell Culture

Umbilical cord derived MSCs and MenSCs were isolated, characterized, cultured and expanded as previously described (Alcayaga-Miranda et al., 2015a; González et al., 2015). Briefly, menstrual blood samples from healthy donors were collected using a silicone menstrual cup (Mialuna^®^, Santiago, Chile). Menstrual blood mononuclear cells were separated by Ficoll-Paque Plus (GE Healthcare, Amersham, United Kingdom) (1.077 g/ml) density gradient according to the manufacturer’s instructions. Cells were collected and cultured in a T25 flask (Falcon^®^, Becton Dickinson, United States) containing Dulbecco’s modified Eagle’s medium (DMEM) high glucose supplemented with 1% penicillin/streptomycin solution (10,000 U/ml / 10,000 μg/ml), 1% amphotericin B (250 μg/ml), 1% L-glutamine (200 mM) (all from Gibco, Paisley, United Kingdom) and 15% fetal bovine serum (FBS) (Lonza, Walkersville, MD, United States) at 37°C, 5% CO_2_ to obtain adherent cells. All MSCs were evaluated in their capacity to differentiate into adipocytes, osteocytes, and chondrocytes by using the StemPro Differentiation Kits (Gibco) in accordance with manufacturer’s instructions. Normal human dermal fibroblast- adult (NHDF-Ad CC-2511) were purchased from Lonza and cultured according manufacture’s protocols.

Immunophenotyping of MSCs was performed by flow cytometry using a FACSCanto II cytometer (BD Biosciences, San Jose, CA, United States) after staining with monoclonal antibodies CD105, CD90, CD73, CD44, HLA-DR, CD34, HLA-ABC, CD19, CD14, and CD45 (all from BD Pharmingen) using standard protocol. The data acquired was analyzed using the FlowJo software V10 (Tree Star, Ashland, OR, United States).

### MSCs Stimulation With Pro-inflammatory Cytokines or Deferoxamine (DFX)

Mesenchymal stem cells at 70% confluency was incubated with IL1β and TNFα (10 ng/ml each) (Peprotech, Rocky Hill, NJ, United States) or 150 μM DFX (Sigma-Aldrich, St. Louis, MO, United States) for 24 h in serum-free DMEM supplemented with 1% penicillin/streptomycin and 1% L-glutamine (all from Gibco) at 37°C with 5% CO_2_. Viability of MSCs was determined by trypan blue solution dye (Gibco) under a phase contrast microscope.

### Conditioned Medium

Mesenchymal stem cells were cultured in the absence (control group) or presence of 10 ng/ml IL1β and 10 ng/ml TNFα (Peprotech) in serum-free DMEM supplemented with 1% penicillin-streptomycin and 1% L-glutamine (all from Gibco). After 48 h, the supernatant was collected and the cellular debris were removed by centrifugation at 500 × *g* for 5 min at room temperature. For negative controls, equal volumes of serum- free DMEM were used. The conditioned medium (CM) was stored at -80°C until use.

### Quantification of Secreted Factors by ELISA

Levels of VEGF, bFGF, IL8, PDGFBB, TGFb1, HGF, and IL6 in MSCs-CM, were detected using duo set ELISA (R&D Systems, Minneapolis, MN, United States) according to the manufacturer’s protocol. Hypoxia inducible factor 1 alpha (HIF-1α) abundance was evaluated in cell lysates using the human HIF-1α ELISA kit (Abcam, Cambridge, United Kingdom) as previously described (Oses et al., 2017).

### Proliferation

Quick Cell Proliferation Assay Kit (BioVision, Milpitas, CA, United States) was used to assess proliferation of MSCs and NHDF-Ad, following manufacturer’s instructions. Briefly, MSCs or NHDF-Ad were cultured (1 × 10^3^/well) in a 96-well plate (Falcon) in a final volume of 200 μl/well of DMEM supplemented with 10% FBS or with CM, respectively. Cell proliferation was quantified by measuring the absorbance (Tecan Reader) of the dye solution at 450 nm at different time points.

### Colony Forming Units

Mesenchymal stem cells were evaluated for frequency of fibroblast colony-forming units (CFU-F) as previously described (Alcayaga-Miranda et al., 2015a; González et al., 2015). CFU-F were evaluated in a serial dilution assay: 25 to 250 cells per well were seeded in a six-well plate (Falcon^®^) and cultivated for 14 days. Cells were fixed in 70% methanol and stained with 0.5% crystal violet (Sigma-Aldrich) in 10% methanol for 20 min. After several washes, colonies formed by more than 50 fibroblast-like cells were counted under a light microscope at low magnification. Results were expressed as CFU/initial number of cells plated.

### T Cell Proliferation Assay

Immunosuppressive capacity of MenSCs in comparison to UC-MSCs was assessed in a T-cell proliferation assay. MSCs, pre-stimulated with 10 ng/ml IL1β and TNFα (Peprotech) (control: no stimulation) were seeded in defined cell numbers in 48 well plates (Falcon) and left to adhere. Peripheral blood mononuclear cells (PBMCs) were isolated from heparinized human peripheral blood samples (healthy donors) using density gradient centrifugation. PBMCs were stained with Cell Trace^TM^ Violet (CTV) (Molecular Probes, Springfield, MA, United States) following manufacturer’s instructions and co-cultured with MSCs (MSC:T-cell ratios 1:5 and 1:10) in RPMI 1640 medium supplemented with 10% FBS, 1% L-glutamine, 1% penicillin/streptomycin (all from Gibco). Proliferation of T-cells was stimulated with phytohemagglutinin (PHA; 15 μg/ml, Sigma-Aldrich). After 72 h, cells were harvested and stained for CD3 and CD4 (BD Biosciences). Samples were analyzed by flow cytometry, and the percentage of CD3+CD4+ proliferative T-cells was determined using FlowJo software V10 (Tree Star, Ashland, OR, United States). PBMCs cultured in medium containing PHA without MSCs and PBMCs cultured in absence of PHA and in presence of MSCs served as controls.

### Real-Time Quantitative Polymerase Chain Reaction (RT-qPCR)

Total RNA was extracted by using the RNeasy kit (Qiagen, Marseille, France) from cultured MSCs (without or stimulated with IL1β and TNFα or DFX) or from harvested wound tissue (mouse). RNA (500 ng) was reverse-transcribed by using superscript II kit (Invitrogen) and qPCR was performed at Stratagene Mx3000P (Agilent Technologies, Santa Clara, CA, United States) with the primers listed in Supplementary Table S1 (Supplementary Information). All values were normalized to GAPDH or b-actin as housekeeping genes and expressed as fold change or relative expression using the 2^-ΔΔ*C*_T_^ formula (Livak and Schmittgen, 2001). Gene ontology analysis, gene functional classification and gene ID conversions were performed using PANTHER version 11: Expanded annotation data from Gene Ontology and Reactome pathways, and data analysis tool enhancements (Mi et al., 2017) using Homo sapiens as reference List and Fisher’s Exact with FDR multiple test correction. The 10 most important biological process were selected according to lowest *p*-value. For this analysis were used the differentially expressed genes obtained from the MSCs, stimulated in absence and presence of pro-inflammatory cytokines, according to the Mann–Whitney *U* test to filter the data by an adjusted *p*-value ≤ 0.05 with a ≥1.5-fold difference.

### Cell Migration Assay

Cell migration capacity of MSCs and NHDF-Ad was evaluated using a Transwell two-chamber cell culture method and Transwell inserts (Corning, Cambridge, MA, United States) with an 8 μm pore size polycarbonate membrane. The upper side of the inserts membrane was coated with 0.1% gelatin (Sigma) in PBS for 2 h at 37°C. The MSCs or the NHDF-Ad at a density of 10,000 or 15,000 cells/100 μl, were seeded in the upper compartment and incubated for 5 and 20 h, respectively. The lower compartment contained 500 μl of MSCs-CM or medium alone as chemoattractant factor or control, respectively. After incubation, non-migrating cells were carefully removed from the upper surface of the insert and washed with PBS. The membrane was fixed and stained with crystal violet as described above. The inserts (lower side) were photographed in five random fields under an inverted bright-field microscope at 10× magnification and percentage of area of migrated cells was determined using Image J analysis software (NIH).

### Mouse Wound Healing Model

C57BL/6 were obtained from Jackson Laboratories (8–10-week-old male/female). Animals were randomly assigned to groups according to experimental design and mouse excisional wound splinting model was generated as described previously (Galiano et al., 2004; Wang et al., 2013). Briefly, under anesthesia (Sevoflurane, Baxter SA, Lessines, Belgium) a 6-mm-diameter sterile biopsy punch (Premier medical, Plymouth, PA, United States) was used on shaved and depilated dorsum to create two full-thickness excisional wounds besides the midline. Subsequently mice received intradermal injections at four different sites around each wound using a 30-G syringe (BD Biosciences) containing a total number of 1.0 × 10^6^ MenSCs in 100 μl saline (MenSCs group) or 100 μl saline only (Control group). A donut-shaped silicone splint (Grace Bio-Labs, Sigma) with a 15 mm diameter was centered on the wound and fixed to the skin using a topical adhesive (Histoacryl^®^, B. Braun, Germany) and interrupted 5/0 poliamide sutures (Dafilon^®^, B. Braun). Finally, the wounds were dressed with Tegaderm (3M, St. Paul, MN, United States) and self-adhering elastic bandage (Coban^®^, 3M). Following surgery, mice received saline and analgesic treatment, and were placed under a warming lamp to recover from the anesthesia. The animals were monitoring daily to assess recovery and to ensure wound area remains covered and sterile. Skin tissue samples were harvested using an 8-mm biopsy punch (Premier medical) at different time points for the following analyses.

### Wound Closure and Angiogenesis Analysis

Individual wounds were recorded using a digital camera at 0, 3, 6, 10, and 14 days after treatment, and the wound areas were measured using Image J software (NIH). The percentage of wound closure was calculated as follows: *(area of original wound - area of actual wound)/area of original wound* × *100*. For angiogenesis, photos of skin biopsies were taken above a transillumination device and pictures were processed with the software VesSeg-Tool^1^ (Schenck et al., 2014). After the digital segmentation of the blood vessels, quantitative data were obtained with the option Image Statistics. The percentage of white coverage was picked up, and the percentage of vessels coverage compared to healthy skin (intern control made for each animal) was calculated as follows: (% white coverage of the wound/% white coverage of healthy skin) × 100.

### Histological Analysis

For histological evaluation, mice were euthanized 14 days after treatment. Wound tissues were harvested, fixed in 10% formalin solution (Sigma-Aldrich), and subsequently embedded in paraffin using standard protocols. Samples were cut into 5-μm sections and stained with hematoxylin-eosin (HE, Sigma-Aldrich) to compare re-epithelization rate and the amount of inflammatory infiltration. The total collagen and collagen III content was determined by Van Gieson (Diapath S.p.A, Bergamo, Italy) and Gomori reticular fiber stain (Sigma-Aldrich). Sample pictures were taken with Olympus CX41 microscope and the quantification was determined using ImageJ software (NIH). Immunohistochemistry was performed to detect CD31 (Rabbit polyclonal 1:50, Abcam, Cambridge, MA, United States) using Dako Envision system (Dako, Carpinteria, CA, United States) according to manufacturer’s recommendations. Immunoreactive sections were visualized with diaminobenzidine (DAB) solution (Dako) and counterstained with hematoxylin. Vascular density was evaluated counting the number of CD31^+^ microvessels per mm^2^ using microscope under 100× magnification. Counting was performed in five different fields per each wound using the Image J software. Histological scoring was performed in a blinded fashion. In **Table 1**, were summarized the criteria for semi-quantitative histological scores, modified from previous reports (Wu et al., 2007; Yew et al., 2011; Pelizzo et al., 2015). Briefly, each slide was given a histological score ranging from 0 to 3 according to the following parameters: re-epithelialization, collagen content, angiogenesis and cell infiltration.

**Table 1:** Criteria for semi-quantitative histological scores.

Score	0	1	2	3
Re-epithelialization	None	Regenerated mono-layered epithelium	Regenerated multi-layered epithelium	Differentiated multi-layered epithelium (stratum corneum)
Collagen content	None	Poor	Moderate	High
Angiogenesis	None	Capillary density poor	Capillary density moderate	Capillary density high
Cell infiltration (inflammatory cells)	None	Few	Fair	Rich


### MenSCs Engraftment and Tracking

Menstrual fluids were labeled with PKH26 Red Fluorescent Cell Linker kit (Sigma-Aldrich) according to manufacturer’s instructions. Labeling efficiency was determined by flow cytometry (>97%), as previously described (Alcayaga-Miranda et al., 2015b). Labeled MenSCs (1 × 10^6^ cells/wound) were resuspended in 100 μl of saline solution and injected intradermally. Animals were euthanized at different time points (Days 0, 3, 6, 10, and 14) and the skin wound samples were recovered. Skin samples were digested 1 h at 37°C with 5 mg/ml of Collagenase type IV (Gibco) and 5 μg/ml of DNase I (Roche, Mannheim, Germany). All samples were passed through a 70-μm nylon cell strainer (BD Falcon) and centrifuged. Skin samples were resuspended in flow cytometry buffer (PBS 1x, 0.5% BSA, 0.01% sodium azide). Percentage of PKH26-positive cells was determined by flow cytometry and data obtained were analyzed using the FlowJo data analysis software. A single-cell suspension of saline or control wounds was used each time for negative controls and gate setting in flow cytometry analysis.

### Statistical Analysis

Data are expressed as mean ± standard error. Mann–Whitney *U* test was used to evaluate the differences between groups. One-way analysis of variance followed by Tukey’s post-test was used for analysis of multiple comparison groups. The number of samples per group (*n*) are specified in the figure legends. Probability value, *p* ≤ 0.05 was considered statistically significant.

## Results

### MenSCs Display a Superior Clonogenic Potential in Comparison With UC-MSCs

**FIGURE 1:**
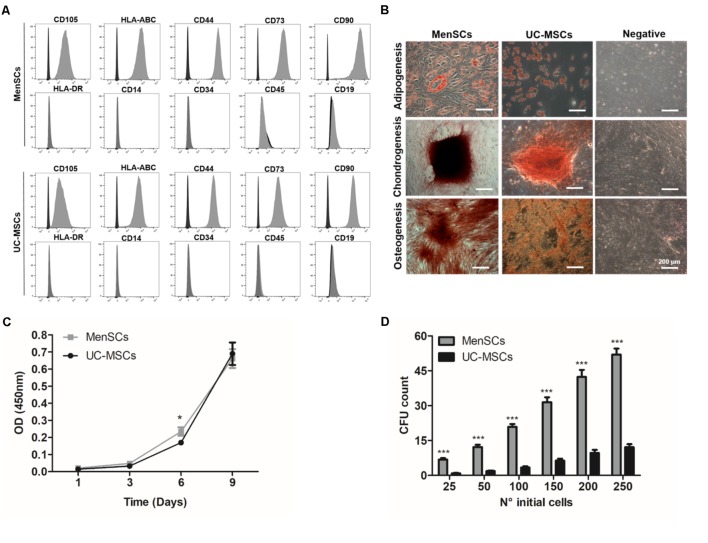
Characterization of MenSCs and UC-MSCs. **(A)** Flow cytometric analysis of MenSCs and UC-MSCs markers. Representative histograms of MSCs surface markers (gray) and their respective isotypes (black). **(B)** MenSCs and UC-MSCs displayed mesodermal tri-differentiation capacity. Representative images of MSCs differentiation after specific inductions and staining to adipocytes (Oil Red O), chondrocytes (safranin O), and osteocytes (alizarin red). Scale bar = 200 μm. **(C)** Proliferation assay of MenSCs and UC-MSCs on days 1, 3, 6, and 9. **(D)** Colony Forming Unit-F assay of MenSCs and UC-MSCs, evaluated until day 14 after cell seeding. Values are expressed as the mean ± SE of at least three independent experiments in triplicates. MenSCs vs. UC-MSCs, ^∗^*p* ≤ 0.05, ^∗∗∗^*p* ≤ 0.001. MenSCs, menstrual derived mesenchymal stem cells; UC-MSCs, umbilical cord mesenchymal stem cells; CFU, colony forming unit; OD, optical density.

All the *in vitro* experiments have been conducted with three different donors of MSCs from the menstrual fluid and umbilical cord tissue, characterized according to their expression of MSCs markers and differentiation potential. Both MSCs showed strong positive expression for CD105, CD90, CD73, CD44 and were negative for hematopoietic cell surface markers including CD34, CD45, CD14, and CD19. Additionally, both MSC sources expressed major histocompatibility complex (MHC) class I antigen HLA-ABC, but not class II HLA-DR (**Figure 1A**). The evaluation of the mesodermal tri-differentiation capacity to osteoblasts, adipocytes, and chondroblasts, showed no differences among cell types (**Figure 1B**). Analysis of proliferative rate over time revealed similar patterns between the different donor tissues, except on day 6 where MenSCs showed an increased proliferation rate (**Figure 1C**). Notably, evaluating the colony forming unit-fibroblast (CFU-F) assay, which reflects the ability of a cell to grow in a density-insensitive fashion (Kurt Yüksel et al., 2010), MenSCs exhibited a notable higher CFU-F potential compared to UC-MSCs. MenSCs initially seeded at ratios 25, 50, 100, 150, 200, and 250 showed an increase of 8-; 6.63-; 6.18-; 4.96-; 4.4-; and 4.2-folds, respectively, compared to UC-MSCs (**Figure 1D**).

### MenSCs Exhibit Higher Immunosuppressive Effects Compared to UC-MSCs Independently of the MSCs Activation State

**FIGURE 2:**
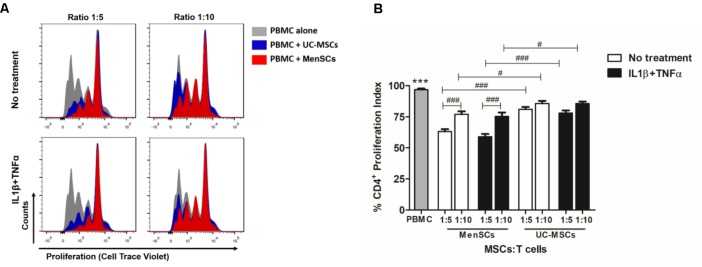
MenSCs display higher immunosuppressive properties resulting in an increased inhibition of T cell proliferation. To study the immunosuppressive capacity of MSCs on T-cell response, we performed an *in vitro* T-cell proliferation assay at different ratios of MSCs:T-cell, as described in Section “Materials and Methods.” MSCs were either unstimulated or were stimulated with IL1β+TNFα (10 ng/ml each) for 24 h before T cells co-culture in complete RPMI medium. Previously, PBMC were labeled with CTV and stimulated with PHA, 15 μg/ml for 3 days. T-cell proliferation was evaluated by flow cytometry, gating on CD3+CD4+ cells. **(A)** Representative histograms of T-cell proliferation in presence and absence of MenSCs and UC-MSCs. **(B)** Graphical presentation of the quantified data. Values are expressed as the mean ± SE of at least 3 independent experiments in triplicates. PBMC alone vs. with MSCs, ^∗∗∗^*p* ≤ 0.001. MSCs vs. each reciprocal condition, ^#^*p* ≤ 0.05, ^###^*p* ≤ 0.001. MSCs, mesenchymal stem cells; IL1 β, interleukin-1 beta; TNFα, tumor necrosis factor alpha; MenSCs, menstrual derived mesenchymal stem cells; UC-MSCs, umbilical cord mesenchymal stem cells; PBMC, peripheral blood mononuclear cells: PHA, phytohaemagglutinin, CTV, Cell trace violet.

The immunosuppressive properties of MenSCs and UC-MSCs were studied by determining their capacity to modulate the proliferative response of CTV-labeled PBMCs upon PHA stimulation. As shown in **Figure 2**, both sources displayed significant immunosuppressive properties in all tested conditions. Interestingly, MenSCs showed an increased effect inhibiting CD4+ T-cell proliferation compared to UC-MSCs at both ratios 1:5 (*p* ≤ 0.001) and 1:10 (*p* ≤ 0.05). We also observed that the inhibitory effect of MenSCs was higher at ratio 1:5 (*p* ≤ 0.001) on the contrary no dose dependent effect was observed for UC-MSCs. To determine if the immunosuppressive effect is maintained in a pro-inflammatory context (as it occurs in injured skin), MSCs were pre-stimulated for 24 h with IL1β and TNFα before co-culture with T-cells. Stimulation with pro-inflammatory cytokines did not affect the ability of MSCs to abrogate T-cell proliferation at the mentioned ratios, and similar as occurred in non-treated cells, MenSCs exhibited a higher inhibition at both ratios (1:5 *p* ≤ 0.001; 1:10 *p* ≤ 0.05) compare to UC-MSCs.

### MenSCs and MenSCs-CM Exhibit Higher Migratory Capacity

**FIGURE 3:**
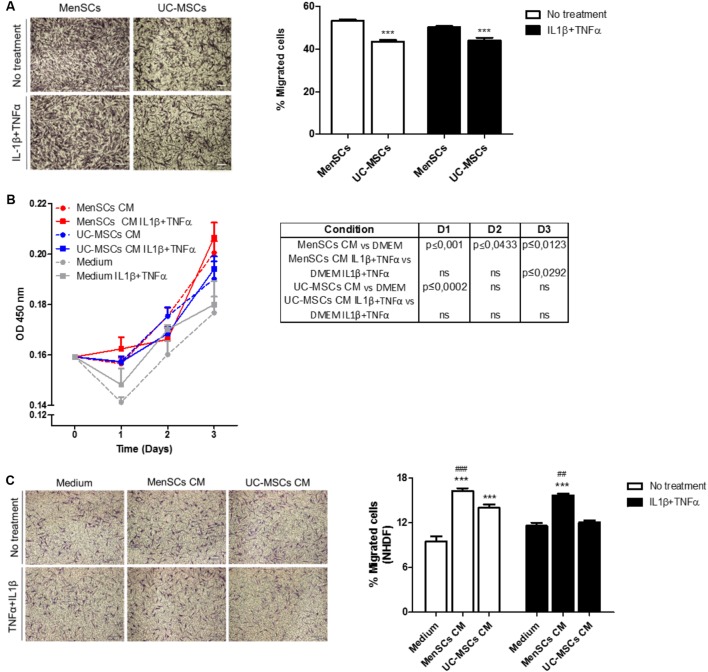
MenSCs and MenSCs-secretome exhibits increased migratory effects. **(A)** Migration of MenSCs and UC-MSCs with and without proinflammatory stimuli. Migration was evaluated by the Transwell assay system. Left panel, representative images of migrated cells. Right panel, quantification of the area of migrated cells per photographic field using Image J software. **(B)** Effect of MSCs-CM on NHDF proliferation. MSCs-CM was prepared by incubating MSCs (2 × 105/well) in serum-free medium or with IL1β and TNFα (10 ng/ml each). Right, table with statistical analysis comparing MSCs-CM vs. Medium. **(C)** Effect of MSCs-CM on NHDF migration. Migration was evaluated by Transwell assay system. MSCs-CM was prepared by incubating MSCs (2 × 105/well) in serum-free medium or with IL1β and TNFα (10 ng/ml each). Left panel, representative images of migrated cells. Right panel, quantification of the area of migrated cells per photographic field using Image J software. Values are expressed as the mean ± SE of at least 3 independent experiments in triplicates. For **(A)** UC-MSCs vs. MenSCs ^∗∗∗^*p* ≤ 0.001 and for **(C)** MSCs-CM vs. Medium, ^∗∗∗^*p* ≤ 0.001; MenSCs-CM vs. UC-MSCs CM, ^###^*p* ≤ 0.001, ^##^*p* ≤ 0.01. CM, conditioned medium; MenSCs, menstrual derived mesenchymal stem cells; UC-MSCs, umbilical cord mesenchymal stem cells; NHDF, normal human dermal fibroblast; TNFα, tumor necrosis factor alpha; IL1β, interleukin 1 beta.

In addition to the immunomodulatory activity, the migratory capacity is an important biological function allowing MSCs to reach and integrate the site of injury and exert their therapeutic effects. Analysis of the migratory capacity revealed significantly increased migration of MenSCs compared to UC-MSCs in both, control conditions and challenged with pro-inflammatory cytokines (*p* ≤ 0.001) (**Figure 3A**). There is several evidence that MSCs secrete paracrine factors that can modulate components of different stages of the wound healing process (Otero-Viñas and Falanga, 2016). Paracrine effects of MSCs on NHD fibroblast (skin cells which play a key role in the ECM production and wound repair) proliferation and migration were investigated, using serum-free CM from respective MSCs cultures (**Figures 3B,C**). Both, MenSCs-CM and UC-MSC-CM enhanced NHDF proliferation at day 1 compared to serum free control medium. However, only MenSCs-CM revealed a persisting stimulatory effect for several days. Interestingly, the CM from stimulated MenSCs showed an increased cell proliferation at day 3 vs. their control medium (**Figure 3B**). Furthermore, both MSCs-CM exhibited a significantly higher chemoattractive effect than their respective control medium on the migration of dermal fibroblast as shown by transwell assay (**Figure 3C**). However, MenSCs-CM showed a greater effect compared to UC-MSCs and this effect is retained with CM obtained from stimulated cells with pro-inflammatory cytokines. Surprisingly, in this case UC-MSCs-CM lost the ability to favor cell migration of dermal fibroblast compared to control medium (**Figure 3C**).

### MenSCs Showed a Differential Transcription Pattern of Genes Associated With Wound Healing

**FIGURE 4:**
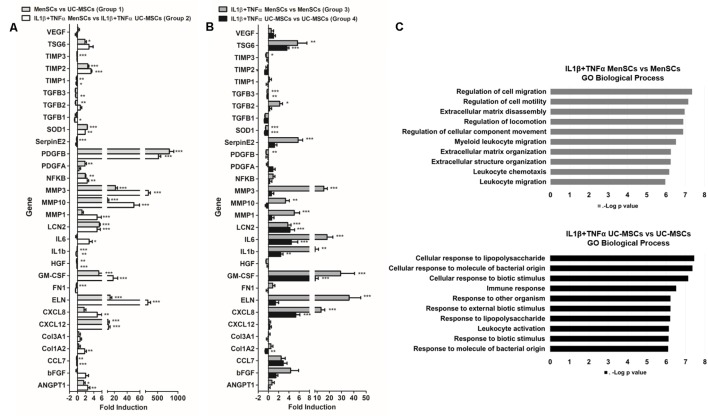
MenSCs exhibited a robust wound healing transcriptomic pattern. MSCs were cultured in basal conditions (medium serum free) and with pro-inflammatory cytokines, IL1β and TNFα (10 ng/ml each) during 24 h. The cells were recovered and total mRNA was extracted, reverse-transcribed and RT-qPCR was performed using Stratagene Mx3000P. The quantitative data from the RT-PCR was normalized by the expression of GAPDH. Expression of different genes involved in wound healing process, **(A)** fold induction of MenSCs vs. UC-MSCs and IL1β+TNFα MenSCs vs. IL1β+TNFα UC-MSCs and **(B)** fold induction of IL1β+TNFα MenSCs vs. MenSCs and IL1β+TNFα UC-MSCs vs. UC-MSCs. Values are expressed as the mean ± SE of at least 3 independent experiments in triplicates. ^∗^*p* ≤ 0.05, ^∗∗^*p* ≤ 0.01, ^∗∗∗^*p* ≤ 0.001 compared to their corresponding MSCs condition. **(C)** GO analysis showing the 10 most important biological process of IL1β+TNFα MenSCs vs. MenSCs and IL1β+TNFα UC-MSCs vs. UC-MSCs. MSCs, mesenchymal stem cells; MenSCs, menstrual derived mesenchymal stem cells; UC-MSCs, umbilical cord-derived mesenchymal stem cells; TNFα, tumor necrosis factor alpha; IL1β, interleukin 1 beta.

An analysis of selected genes involved in wound repair, such as growth factors, cytokines, chemokines, antimicrobial peptides, metalloproteinases (among others), was performed to investigate the relative mRNA expression levels, in both, basal and in pro-inflammatory conditions. As shown in **Figure 4**, we analyzed 30 genes and compared four groups of data: Group 1: MenSCs vs. UC-MSCs; Group 2: IL1β+TNFα MenSCs vs. IL1β+TNFα UC-MSCs; Group 3: IL1β+TNFα MenSCs vs. MenSCs, and Group 4: IL1β+TNFα UC-MSCs vs. UC-MSCs.

Our results indicated that in basal conditions, the expression levels of several genes were considerably different between MSCs, and these differences became more pronounced when the cells were treated with pro-inflammatory cytokines IL1β+TNFα (**Figures 4A,B** and Supplementary Table S2). In particular, without treatment, a significant up-regulation of 43% and a down-regulation of 27% of genes in MenSCs was observed in comparison with UC-MSCs (**Figure 4A**, Group 1). More in detail, genes involved in angiogenesis and neovascularization ANGPT1 (1.5-fold), PDGFA (1.8-fold), PDGFB (791-fold), and MMP3 (21.6-fold), ECM components ELN (13.4-fold) and MMP10 (9.2-fold) which helps to control degradation of collagen in the wound during tissue remodeling (Wang et al., 2012; Yolanda, 2014), showed a significantly up-regulation in MenSCs compared to UC-MSCs. Furthermore, genes implicated with anti-apoptotic and anti-fibrotic properties, and immunomodulation, anti-microbial effects and transcriptional pathways like GM-CSF (5.2-fold) and TIMP2 (2.5-fold), TSG6 (1.8-fold), LCN2 (5.6-fold) and NFkB1 (1.9-fold), respectively, were also increased in MenSCs compared to UC-MSCs. By contrast, a down-regulation of TGFB2 (0.47-fold) was observed, factor which higher activity was related to keloids (Xia et al., 2006). Interestingly, MenSCs compared to UC-MSCs expressed lower but significant levels of SERPINE2 (0.19-fold) and IL1β (0.09-fold), the former encoding a protein that inhibit serine proteases and displays anti-angiogenic function (Bhakuni et al., 2016) and the second the pro-inflammatory IL1β (**Figure 4A**, Group 1). The pro-inflammatory condition significantly regulated differentially the mRNA expression of 53% of the genes in MenSCs (**Figure 4B**, Group 3) while only 33% in UC-MSCs (**Figure 4B**, Group 4). Collectively these results suggest that MenSCs are more sensitive to an inflammatory environment. Among the genes identified as responding to the stimuli (73.3%) in MenSCs vs. UC-MSCs (**Figure 4A**, Group 2), it can be found that PDGFB (523-fold), MMP3 (274-fold), ELN (261-fold), and MMP10 (51-fold) displayed the highest increase. Other genes that displayed a significant expression increase were IL8 (4.9-fold), a potent angiogenic and inflammatory chemokine that up-regulates VEGF expression (Martin et al., 2009); bFGF (2-fold), also a pro-angiogenic factor that influences granulation tissue formation, epithelialization, and tissue remodeling (Eming et al., 2014); and IL6 (2.9-fold), a pro-inflammatory cytokine that has notable effects in immunomodulation and on angiogenic processes as promoting endothelial progenitor cell migration and proliferation, regulation of bFGF, induction of PDGF as well as stimulation of vascular smooth muscle cell migration (Kang et al., 2015). GO analysis were used to identify at least the 10 most important biological processes in which the differentially expressed genes in stimulated MSCs were involved (**Figure 4C**). This data suggests that in pro-inflammatory conditions MenSCs will be associated with tissue remodeling and UC-MSCs with activators of the immune system.

### Hypoxia Condition Increases the Expression of Pro-angiogenic Factors

**FIGURE 5:**
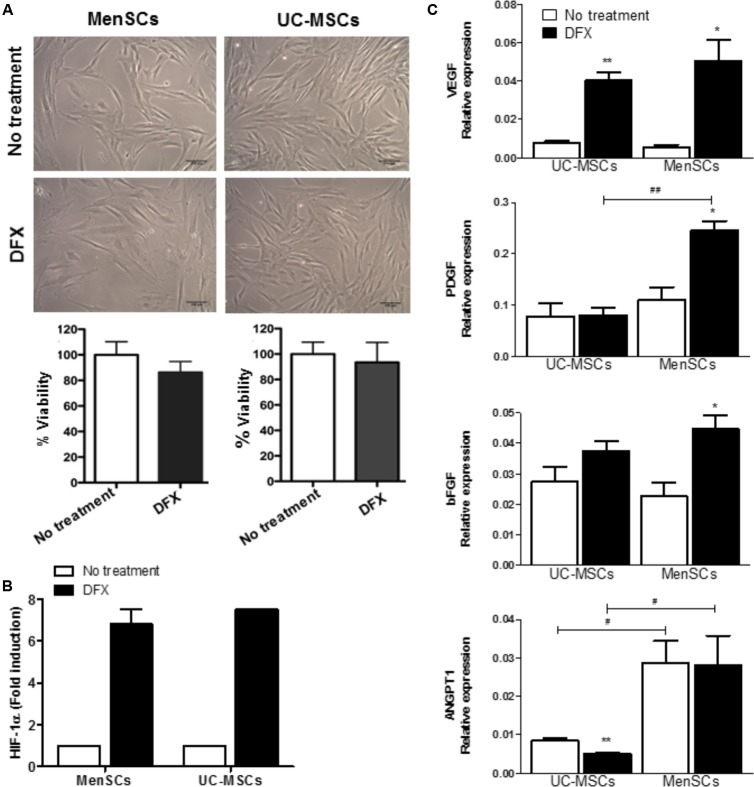
Increased expression levels of pro-angiogenic factors in hypoxia conditions. MSCs were incubated during 24 h with 150 μM of DFX. **(A)** Upper: Representative images showing no changes of morphological characteristics of MSCs comparing to no treated control (without DFX). Lower: Percentage of MSCs viability quantified by trypan blue exclusion assay. **(B)** Levels of HIF-1α determined by ELISA in MSCs lysates. Data were expressed as fold change comparing to non-treated control (without DFX). Values are expressed as the mean ± SE. **(C)** Expression of mRNA levels of the pro-angiogenic factors VEGFα, ANGPT1, PDGF, and bFGF. Values are expressed as the mean ± SE. Treated DFX MSCs vs. non-treated MSCs, ^∗^*p* ≤ 0.05, ^∗∗^*p* ≤ 0.01. MenSCs vs. their respective condition in UC-MSCs, ^#^*p* ≤ 0.05, ^##^*p* ≤ 0.01. MSCs, mesenchymal stem cells; MenSCs, menstrual derived mesenchymal stem cells; UC-MSCs, umbilical cord-derived mesenchymal stem cells; DFX, deferoxamine; HIF-1α, hypoxia inducible factor 1 alpha; VEGF, vascular endothelial growth factor; ANGPT1, angiopoietin 1; PDGF, platelet derived growth factor; bFGF, basic fibroblast growth factor.

Hypoxia, inflammation, infection, arterial and venous insufficiency are part of the non-healing wound microenvironment (Chae et al., 2017). Therefore, we decided to evaluate in both MSCs the differential expression of pro-angiogenic genes during hypoxia induced by deferoxamine (DFX), a hypoxia mimetic agent. A concentration of 150 μM of DFX was selected since it has been previously reported that this dose improves their therapeutic potential with no toxic effects detected on MSCs (Oses et al., 2017). The treatment with DFX revealed no changes in morphology or viability of both, MenSCs and UC-MSCs as shown in **Figure 5A**. DFX induced an increase of HIF-1α levels in MenSCs (6.82-fold) and UC-MSCs (7.5-fold). HIF-1α is a factor known for activating the transcription of pro-angiogenic genes (**Figure 5B**). Under DFX culture condition, MenSCs showed a significant increase of VEGF (9.96-fold), PDGF (2.22-fold), and bFGF (1.95-fold) mRNA expression levels. In contrast, among factors expressed by UC-MSCs only VEGF experienced a 5.35-fold increase. Comparing differences in the expression levels of factors between the two cell sources under hypoxic conditions, only ANGPT1 and PDGF secreted by MenSCs showed an increased expression of 5.58 and 3.07-fold, respectively (**Figure 5C**).

### MenSCs on the Healing Milieu Promotes Changes in the Matrix Signal and Enhance the Angiogenic Process *in Vivo*

**FIGURE 6:**
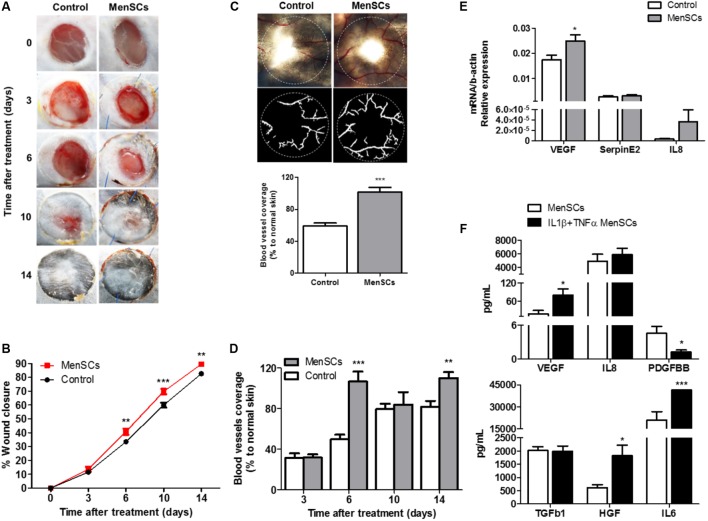
MenSCs injection accelerate the wound healing process in a mouse excisional wound splinting model. Percentage of wound closure and angiogenesis were evaluated after the mice were injected (intradermal) with 1 × 106/100 μl of MenSCs (MenSCs group, *n* = 49) or Saline (Control group, *n* = 38). **(A)** Representative images of the silicone-splinted excisioned wound at different time points. **(B)** Percentage of wound closure at different time points. The percentage of wound closure was calculated using the Image J Software. **(C)** Representative images and quantification of angiogenesis in skin biopsies 14 days after treatment. The photos were taken above a transillumination device (upper photos) and the digital segmentation (lower photos) of the blood vessels and angiogenesis quantification was performed using the VesSeg-Tool software. **(D)** Quantification of angiogenesis in skin biopsies at different time points, analyzed with the software VesSeg-Tool. **(E)** Quantitative RT-PCR to determine expression levels of key genes associated with angiogenesis process in skin biopsies 14 days after injection. Values represent mean ± SE. MenSCs group vs. Control group,^∗^*p* ≤ 0.05, ^∗∗^*p* ≤ 0.01, ^∗∗∗^*p* ≤ 0.001. **(F)** Levels of secreted angiogenesis and repair mediated factors measured by ELISA in MenSCs supernatant in the absence or presence of pro-inflammatory stimuli, IL1β+TNFα for 24 h *in vitro*. Values are expressed as the mean ± SE of at least 3 independent experiments in triplicates. IL1β+TNFα MenSCs vs. MenSCs, ^∗^*p* ≤ 0.05; ^∗∗∗^*p* ≤ 0.001, menstrual derived mesenchymal stem cells; TNFα, tumor necrosis factor alpha; IL1β, interleukin 1 beta; VEGF, vascular endothelial growth factor; IL8, interleukin 8; PDGFBB, platelet derived growth factor BB; TGFb1, transforming growth factor beta 1; HGF, hepatocyte growth factor; IL6, interleukin 6.

MenSCs were directly injected into the 6-mm diameter wound sites of animals from the mouse excisional wound-splitting model. As shown in **Figure 6A**, macroscopical changes started to be perceived at day 6 in the field surrounding the open wound. There were significant differences in the percentage of wound closure among the groups during the 14-day follow-up period. Quantitative analysis showed that the measured wound closure significantly increased in mice treated with MenSCs starting from day 6 and persisted until the 10- and 14-day post-injection (**Figure 6B**), indicating an acceleration of the wound closure. The vascularization of skin biopsies was detected by direct blood vessel visualization and digital segmentation. At the end point, MenSCs promote an increased formation of a well-defined vascular network when compared to control group (**Figure 6C**). The vascularization reached values of 41.8% significantly higher than in control group. Notably, the quantification of the vascularization analyzed at different time points indicated an enhanced network by MenSCs statistically significant starting at day 6 with a 53.42% increase over the control. That was maintained, but at lower rates until day 14, suggesting a faster neovascularization with MenSCs treatment (**Figure 6D**). To determine the specificity of the MenSCs effect, human dermal fibroblasts were administered in an additional experimental group. As expected, the fibroblast injection showed no effect on the wound closure rate and angiogenesis (Supplementary Figure S1).

**FIGURE 7:**
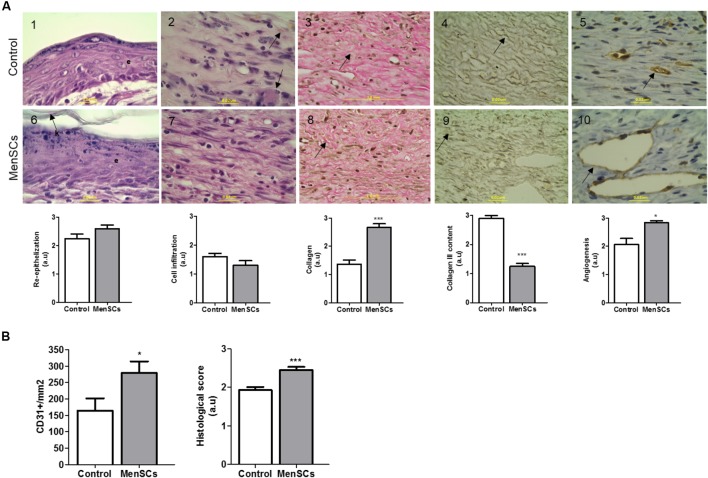
Histologic analysis of wound sections in the wound healing model. For histological evaluation, mice were euthanized 14 days after treatment (Saline or MenSCs) and the wound tissues were harvested. **(A)** Upper panel, representative images of each parameter of Control group (1–5); Lower panel, representative images of each parameter of MenSCs group (6–10). Re-epithelialization evaluated by HE staining (Panels 1,6), arrow indicates keratin layer (k) and e, epithelial layer; Cell infiltration evaluated by HE staining (Panels 2,7), arrow indicates macrophages presence in Control group; Collagen content evaluated by Van gieson stain (Panels 3–8), arrow indicate the collagen bundles, compact in treated group; Collagen III content evaluated by silver stain Gomori (Panels 4,9), arrow indicates collagen III fiber, in treated group fibers are more disorganized, shorter an thinner than control; Angiogenesis evaluated by immunohistochemistry with anti-CD31 (Panels 5,10), arrow indicate the vessel stain with CD31. Quantification of CD31+ was performed using Image J software. (100×, scale bars 2 mm or 0,2 μum as shown in image). Below photos, semi-quantitative score of each parameter. **(B)** Quantification of CD31+ using Image J software (left panel). Semi-quantitative total histological score of MenSCs group (*n* = 14) comparing to Control (*n* = 14) (right panel). Values represent mean ± SE, MenSCs group vs. Control, ^∗^*p* ≤ 0.05, ^∗∗∗^*p* ≤ 0.001. MenSCs, menstrual derived mesenchymal stem cells; HE, hematoxylin-eosin; a.u., arbitrary units.

To gain insight on the factors involved in the enhanced reparative effect of MenSCs and their induction by the wound site milieu, the relative mRNA expression of endogenous VEGF, IL8 and Serpine2 were measured in skin biopsies at day 14 (**Figure 6E**). The data showed a significant up-regulation of VEGF levels in the MenSCs treated tissues compared to the control, IL8 showed a tendency to increase while Serpine2 remained unchanged. These results were consistent with the histological findings by IHC staining, where higher significant differences in the CD31 angiogenesis marker were observed in the treated group with 278.63 CD31^+^/mm^2^ vs. 163.85 CD31^+^/mm^2^ in control (**Figure 7**, panels 5,10). In MenSCs group, there was a high quantity of blood vessels lumens of different diameters distributed throughout the dermis in a homogeneous manner and large lymphatic vessels predominantly present in the lower part of the dermis. In contrast, the saline group sections displayed a lower number of blood vessels of diameters mostly smaller (**Figure 7**, panels 5,10). On the other hand, we determined the accumulation of pro-angiogenic factors in MenSCs-CM by ELISA with the presence or absence of the pro-inflammatory stimuli, to address the probable contribution of these factors in the healing process (**Figure 6F**). Detectable levels of VEGF, IL8 and PDGF-BB were found in both conditions. However, in the presence of pro-inflammatory cytokines, a significant increase in VEGF secretion was observed, but conversely, the levels of PDGF-BB decreased with the stimuli. Additionally, IL8 although abundant, did not experience significant changes. Also, we determined the accumulation of several growth factors, cytokines or ECM components. Interestingly, IL6 and HGF release were higher in pro-inflammatory conditions while TGFβ1 level remained unchanged.

### MenSCs Treatment Improves the Quality of the Repaired Cutaneous Tissue

Consequently, we evaluated the benefit of the treatment with MenSCs on epidermal healing by analyzing the re-epithelialization of the wound site. HE staining showed in most cases that MenSCs-treated wounds had advanced re-epithelization (multi-layered) with presence of keratin formation compared to control, although this observed difference did not become significant (**Figure 7**, panels 1,6). Nonetheless, skin appendages, i.e., hair follicles, sweat glands and sebaceous glands, were not observed in any of the group of animals. Taking into account that the inflammatory phase occurs within the first 3 days of wound healing, low levels of inflammatory cell infiltration were detected in both groups, with a tendency of less infiltration in the treated group (**Figure 7**, panels 2,7). This observation correlates with the immunosuppressive properties described in the *in vitro* setting.

**FIGURE 8:**
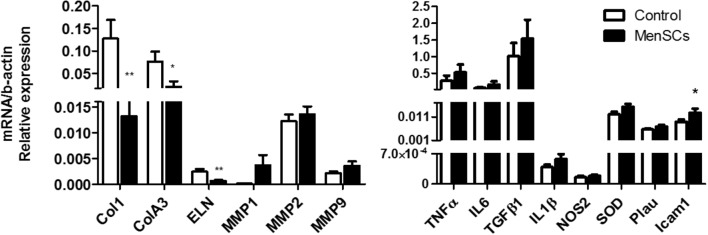
Expression of matrix signal related genes from the wound site. Quantitative RT-PCR analysis was performed in skin biopsies harvested 14 days after treatment (Saline and MenSCs). Expression of genes associated with wound healing was analyzed. The quantitative data from the RT-PCR was normalized by the expression of b-actin. Values are expressed as the mean ± SE. MenSCs vs. control group, ^∗^*p* ≤ 0.05, ^∗∗^*p* ≤ 0.01. MenSCs, menstrual derived mesenchymal stem cells.

To evaluate the quality of the repair, the deposition and organization of collagen, central component of ECM products, were analyzed. The collagen fibers content was very compact, dense and significantly increased in the reticular dermis of MenSCs-treated wound compared to the control group as showed by Van Gieson’s staining (**Figure 7**, panels 3,8). Furthermore, the amount of organized collagen (birefringent), revealed by polarized light microscopy on the same slides was also higher in the treated group (not showed). In the MenSCs injected group, the wound area contains dense thick collagen bundles arranged in diverse directions and parallel to the surface as observed in contiguous normal skin. On the other hand, the control group showed a wound with dense but thinner collagen bundles displayed in a less organized pattern (**Figure 7**, panels 3,8). In addition, with silver staining we determined the reticular fibers made up of thin bundles of fine type III collagen fibrils. As seeing in **Figure 7**, panels 4,9 there is a significant lower content of type III collagen in treated wound compared to controls. Type III collagen represents the firstly formed more disorganized fibers and type I collagen represents later formed and more organized fibers suggesting that the healing process was accelerated in the MenSCs treated wound. The healing index (semi-quantitative score parameters are shown in **Table 1**) of wounds treated with MenSCs was significantly greater than those treated with saline (**Figure 7B**). Interestingly, we also investigated in wound tissues the mRNA expression of several factors and cytokines known to be associated with the wound healing process. The results revealed significant differences in the expression of genes related to ECM remodeling such as Col1, ColA3, ELN, or Icam1, when comparing both groups (**Figure 8**). The general conclusion is that the MenSCs treatment increase the rate of wound closure, forming a robust vascular network with dense collagen content promoting proper tissue regeneration.

### Fate and Distribution of Engrafted MenSCs

**FIGURE 9:**
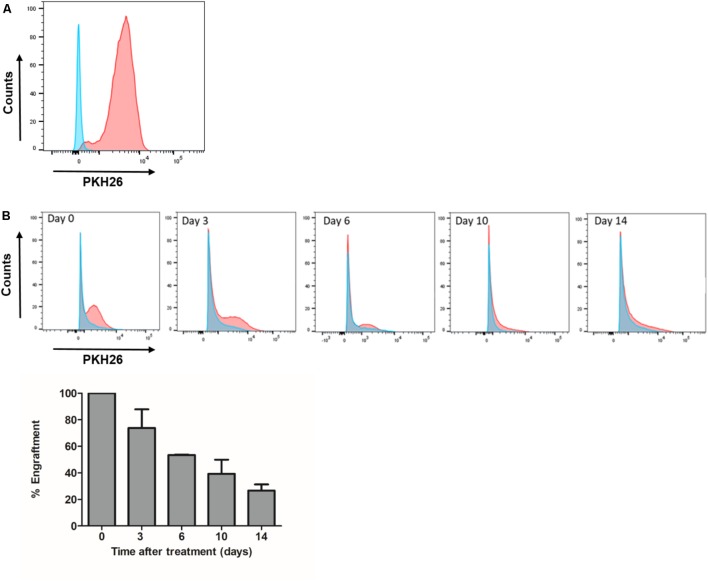
Engraftment of MenSCs into the wound persist for at least 2 weeks of treatment. Each wound received an intradermal injection of one million total MenSCs labeled with PKH26 and an equal volume of saline (Control group). **(A)** Representative plot of labeled MenSCs showing 97% of labeling efficiency (red line). **(B)** The engraftment at different time points was evaluated by FACS analyses of wound digests for PKH26 positive MenSCs. Cells from control wounds were used for negative controls and gate setting (blue line). Percentages of PKH26 positive cells are indicated. Values are expressed as the mean ± SE. MenSCs, menstrual derived mesenchymal stem cells.

To examine the persistence of intra-dermally administered MenSCs at the wound site, we labeled the cells with PKH26 dye prior to their injection. The labeling efficiency of MenSCs with PKH26 before injection was found to be >97% (**Figure 9A**). At different time point post-injection, the entire wound and skin included in the splint were enzymatically dispersed into a single cell suspension and analyzed by flow cytometry. The absolute number of MenSCs obtained at the same day of the injection (day 0) was set as 100% of the engraftment. The percentages of engrafted MenSCs were obtained at different times of 3, 6, 10, and 14 days after injection. A gradual decrease in the percentage of fluorescent positive cells was observed started at day 3 (73.8%) and persisted throughout the experiment. We were able to detect PKH26+ cells until the endpoint of the experiment, at day 14 with 26.6% of the initial engraftment (**Figure 9B**).

## Discussion

Although bone marrow, adipose tissue, and umbilical cord are well established sources of MSCs used in clinical trials to treat skin ulcers^2^, they are prone to a number of disadvantages, which MenSCs can easily overcome: non-invasive and repetitive isolation procedure, as well as high frequency of mesenchymal progenitors and proliferative rate that allow a clinical-scale allogeneic transplantation in less time and recurrent applications (; Meng et al., 2007; Alcayaga-Miranda et al., 2015a). The efficacy of MSCs from various sources showed discrepancies, and as a consequence extrapolation of results from one source to other MSCs types would suffer from inaccuracy (Akimoto et al., 2013; Lee et al., 2016). In this study we demonstrate for the first time that MenSCs through the enhanced properties presented here, can improve and accelerate the regenerative process in wound healing, promoting MenSCs as strong candidates for the treatment of skin ulcers.

In the *in vitro* comparative study with UC-MSCs, different classical and functional properties related to the repair process were evaluated. While no difference in the immunophenotypic analysis or differentiation capacity was noted, MenSCs showed a superior clonogenic capability compared to UC-MSCs. The relevance of a higher CFU indicate the capacity of cells to grow in a density-insensitive fashion, an important attribute to increase the quality of the newly regenerated skin. One of the mechanism implicated in the repair abilities of MenSCs is their high migration capacity to injury sites in response to signals of cellular damage. In this report, under both basal and pro-inflammatory conditions, MenSCs demonstrated an increased migration ability respect to UC-MSCs. Consistent with this data, it has been shown that in presence of a potent inflammatory stimulus, e.g., bacterial lipopolysaccharide, MenSCs display a strong migration potential, however, further *in vivo* studies are required to fully address this property. In previous reports, MenSCs also outmatched other sources such as ADSCs or BM-MSCs (Alcayaga-Miranda et al., 2015a,b; Chen et al., 2015; Fathi-Kazerooni et al., 2017).

One of the most important challenges for the treatment of non-healing wounds is the presence of sustained inflammation and infections (Zahorec et al., 2015; Otero-Viñas and Falanga, 2016). In this regard, MSCs have been demonstrated to exhibit several antimicrobial and immunomodulatory effects on host immune cells (Luz-Crawford et al., 2016b; Harman et al., 2017; Johnson et al., 2017). Analyzing the immunosuppressive effect of both MSCs, we found that MenSCs exhibit an increased inhibition on CD4+ T cell proliferation than UC-MSCs, at 1:5 and 1:10 cell ratio. Moreover, in contrast to UC-MSCs, MenSCs were more efficient to inhibit T proliferation at ratio 1:5. Interestingly, our group previously demonstrated that MenSCs showed similar immunosuppressive effect as BM-MSCs at 1:10 ratio but fail to match them when diluted to a 1:100 ratio (Luz-Crawford et al., 2016b). In agreement with that, Nikoo et al. (2012) described that MenSCs exert their immunoregulatory activity in a dose-dependent manner. In summary, both MSCs origin and dosis are directly implicated in the changes of immunosuppressive properties of MSCs. These variables are important requisites, which need to be considered for consistent clinical outcomes. In injured sites, the recruited MSCs can be activated by the secretome of activated lymphocytes and monocytes, or by combinations of IFNγ with TNFα, IL1α, or IL1β (Ren et al., 2008). Thus, to functionally define the impact of a pro-inflammatory environment in the suppressive signature of MenSCs, we performed the T cell proliferation assay with MSCs activated with pro-inflammatory cytokines.

Similar to the basal conditions, the experimental outcome pointed at a higher inhibition of T cell proliferation of activated MenSCs when directly compared with UC-MSCs. This suggest that the immunosuppressive potential would be conserved independently of the pro-inflammatory stimuli.

Paracrine factors have been described to play a key role in the mechanisms accounting for tissue regeneration. Multiple angiogenic, growth and trophic factors, as well as chemokines, cytokines, hormones, and extracellular matrix proteins have been identified as constituents of the MSCs secretome (Walter et al., 2010; Figueroa et al., 2014). There are several studies demonstrating the benefit of the conditioned medium derived from MSCs in wound repair (Yew et al., 2011; Akram et al., 2013). To gain insight in the mechanism behind it, we further investigated the selected biological function and identified a specific transcriptomic pattern of relevant factors known to play an essential role in the different pathways involved in wound healing. The recruitment of fibroblasts for instance, is directly involved in the wound healing process, contributing to the deposit of collagen and other ECM proteins and hence, remodeling the immature collagen matrix (Tracy et al., 2016). The experimental results presented here, show that MenSCs-CM induced an increased migration of fibroblasts compared to UC-MSCs CM under basal and pro-inflammatory conditions. Moreover, both MSCs-CM showed an induction of the proliferation rate compared to medium alone, and this effect was independent of the pro-inflammatory stimulation of MSCs. Several studies have disclosed similar results, showing an enhanced dermal fibroblast migration and proliferation using conditioned media from UC-MSCs (Walter et al., 2010; Stessuk et al., 2016; Li et al., 2017). Among the factors that stimulate increased cell migration in dermal fibroblasts, TGFb1, and IL6 appears to be essential elements (Postlethwaite et al., 1987; Luckett and Gallucci, 2007). Those factors were both expressed by MenSCs and UC-MSCs as we and others showed by qPCR and ELISA (Luz-Crawford et al., 2016a; Chen et al., 2017). This finding, in addition, with the observed immunosuppressive properties, suggests that the mediators secreted by MenSCs could affect positively the local microenvironment, which would facilitate proliferation, recruitment and migration of resident cells as a response to chemoattractants and may reinforce tissue regeneration and repair.

In an inflammatory environment, MSCs have been shown to display an anti-inflammatory phenotype, characterized by an increased expression of IL6, among other cytokines (Bernardo and Fibbe, 2013). In line with these findings, we analyzed differential expression of genes associated to the wound healing process in MenSCs and UC-MSCS, in basal and pro-inflammatory conditions Overall, 70–73% of the analyzed genes differed in expression between MenSCs vs. UC-MSCs in both conditions. When subjected to pro-inflammatory stimuli, MenSCs altered expression of 53% of analyzed genes, while UC-MSCs only changed 33%. This suggests that MenSCs are more responsive to an inflammatory environment. Notably, within the stimulated MenSCs, genes as MMPs, ELN, PDGF, IL8, IL6, and IL1β were strongly upregulated in comparison with stimulated UC-MSCs. It is interesting to note that- although there are no differences between MenSCs and UC-MSCs- both increased their expression of LCN2 in response to the pro-inflammatory cytokines. LCN2 is an antimicrobial peptide (AMP), upregulated at sites of inflammation and injury (Alcayaga-Miranda et al., 2017). It has been recently described in mouse BM-MSCs secretome as well as in equine MSCs culture, that it is at least partially responsible for the antimicrobial effects described for MSCs (Gupta et al., 2012; Harman et al., 2017). Additionally, it has been described that LCN2 has a crucial role in promoting epidermal cell migration and wound healing in skin (Miao et al., 2014). To our knowledge, LCN2 has not been reported to be expressed in UC-MSCs or MenSCs, hence, this is the first study to report the expression of this AMP. It would be of high interest to investigate further understand the effect of LCN2 in a wound infection model. Analysis of the biological processes with the Gene Ontology, revealed that the genes differentially expressed in stimulated MenSCs can be associated with tissue remodeling and in UC-MSCs with activators of the immune system. Wegmeyer et al. (2013) using pathway analysis of gene expression data, found that UC-MSCs possess a higher immunomodulatory potential compared to BM-MSCs, demonstrating a functional diversity between MSCs from distinct sources. Non-healing wounds are associated to a hypoxic state. We studied this condition in MenSCs and UC-MSCs, using a hypoxia mimic agent (DFX). The treatment induced an increase in HIF-1α levels, which in turn promoted the overexpression of genes related to pro-angiogenic responses. Indeed, DFX-stimulated MenSCs displayed an enhanced angiogenic potential, paralleled by an increased expression level of factors such as VEGF, PDGF, and bFGF while in UC-MSCs only VEGF augmented. Comparing treated MenSCs with UC-MSCs, VEGF, and PDGF showed the highest up-regulation. These results are in agreement with previous reports obtained with MSCs from adipose tissue or bone marrow (Potier et al., 2008; Oses et al., 2017). Altogether, these data corroborate that MenSCs and UC-MSCs sense the microenvironment and display different pattern of expressed molecules according to the particular need of injured tissue.

Given that MenSCs share features with UC-MSCs that in some cases were found to be superior, and the ample published reports demonstrating the beneficial effect of UC-MSCs on wound healing (Shrestha et al., 2013; Pereira et al., 2016), we investigate the effect of MenSCs on the signal molecules in the wound site and assayed the therapeutical outcome of this treatment in a well described excisional wound splinting mice model (Galiano et al., 2004). Our data indicated that the injection of MenSCs intradermally favored wound healing. The wound closure and angiogenesis of the treated group progressed more rapidly than the control indicating an acceleration in healing process. These results were consistent with those obtained for other MSCs sources such as UC-MSCs or BM-MSCs (Wu et al., 2007; Fong et al., 2014). Revascularization of the wound bed is a crucial step of the normal wound healing process, where new vessels form as granulation tissue develops to supply blood to the wound area, which requires oxygen and nutrients (Nuschke, 2014). The results showed that MenSCs can promote an increased formation of a well-defined vascular network extended in the wound compared to the control group, suggesting that MenSCs promotes angiogenesis. These differences determined by direct blood vessel visualization and confirmed with immunohistological staining with CD31, was notorious from day 6 after treatment showing an increase which correlates with the observed acceleration of wound closure, until the endpoint of 14 days. We determined that MenSCs treatment also increased the mRNA expression levels of endogenous pro-angiogenic factors such as VEGF and IL8 in the mice wounds. These results are consistent with the *in vitro* data of the MenSCs-CM showing high levels of VEGF and IL8. Furthermore, the secreted levels of VEGF were elevated in response to pro-inflammatory stimuli. VEGF, in the wound bed, is known to play a key role in angiogenesis, stimulating endothelial proliferation, migration and increased circulation endothelial progenitor cells (Herrmann et al., 2011; Nuschke, 2014). IL8 stimulates angiogenesis through enhancement of endothelial cell survival, and proliferation (Li et al., 2003). Interestingly, factors that regulate VEGF and IL-8 such as TGFb1, HGF, and IL6 appeared to be highly secreted by MenSCs, and increased following the pro-inflammatory stimulation. Along with a greater maturation of the vascularization of the wound, the MenSCs-treated group displayed a highly increment in density and content of collagen. The collagen fibers in the MenSCs group were well organized in dense thick bundles while in the control group there were thinner and less organized. Moreover, the lower collagen III quantity detected in the treated mice strongly suggest an accelerated and therefore more mature remodeling process in contrast to group injected with saline, since this collagen is the firstly formed in wound healing process (Landén et al., 2016). The main cell type involved in wound remodeling and responsible for collagen deposition are dermal fibroblasts (DF). An increased presence of DF was detected in the MenSCs treated wound niche as revealed by the intense cellularity detected in the histology sections. Our qRT-PCR analysis, showed that MenSCs in pro-inflammatory conditions up-regulate gene expression of fibronectin, elastin, collagen and MMPs, indicating a contribution to the wound remodeling. In previous work it has been demonstrated that MSCs produce MMPs, collagen types I and III providing long term reconstruction of the wound (Fathke et al., 2004; Almalki and Agrawal, 2016). As mentioned before, MenSCs secreted factors such as HGF, IL6, IL8, and TGFb1 which in turn have been shown to prevent apoptosis and promote keratinocyte and fibroblast migration and proliferation (Gallucci et al., 2004; Nishikai-Yan Shen et al., 2017). Therefore, these factors can actively participate in the observed improved wound healing in the treated group. The modulatory effect of MenSCs on different matrix signals was revealed by qRT-PCR on wound biopsies. We observed that the MenSCs treatment up-regulates the expression of mouse ICAM-1 and VEGF compare to control group, suggesting that they may be producing adhesion molecules for the acceleration of wound closure. In contrast, the expression of Col1A, Col3A and ELN in the treated wounds was down-regulated. This may be explained by a more advanced stage of the wound healing processes in the MenSCs group, in contrast to early stages, where continuous synthesis of collagens and elastin is still required. Finally, using PKH26 labeling technics, we observed a considerable engraftment of MenSCs at days 3 and 6 after treatment with a rapid reduction at day 14. Consistent with our findings, Wu et al. (2007) also observed with GFP-expressing BM-MSCs a diminished percentage over time in a similar wound healing model (Leibacher and Henschler, 2016). MenSCs presence at the wound site for at least 2-week post-transplantation is a good indicator of a persisting beneficial roles covering the different stages of the healing process.

While we report here, a promising potential for the application of MenSCs in wound injuries, it is still necessary to complete their preclinical characterization with further experimental models including chronic wound models related to diabetes and/or infections. Moreover, is still needed a better mechanistic understanding of MenSCs, along with studies to identify the specific interactions between MSCs, their paracrine factors and the other cell types present in the wound microenvironment.

All these results taken together point at enhanced reparative abilities of MenSCs assigned to a combination of biological properties including extensive immunosuppressive and paracrine effects that are highly responsive to the wound environment. These attributes can modulate the wound matrix signals, hence, contributing to an improved cutaneous regeneration. Besides their detailed therapeutical potential, MenSCs have important practical advantages over other MSC origins that convert them into an important alternative source for cell therapy. Indeed, these cells are devoid of ethical problems, have non-invasive isolation and expansion procedures, additionally, they are well tolerated since there have been no reports of toxicity or side effects (Khoury et al., 2014). In fact, the first report of clinical application of MenSCs involved the allogenic injection of four patients with Multiple sclerosis, showing no apparent adverse effects (Zhong et al., 2009). Also, a stem cell company, launched a phase II clinical trial with MenSCs for congestive heart failure, with patients receiving escalating doses up to 200 million cells from a universal donor (Bockeria et al., 2013). Moreover, FDA clearance was obtained for the treatment of critical limb ischemia, an advanced form of peripheral artery disease (Khoury et al., 2014).

## Conclusion

The study shows that MSCs from different sources display distinct biological properties, which may be relevant for clinical application of different MSCs types for treatment of specific diseases. The different biological functions including, the superior clonogenicity, immunosuppressive and migratory properties in combination with specific gene/paracrine signature of MenSCs resulted in an enhanced wound healing and cutaneous regeneration process *in vivo*. In addition, our findings provide evidence that the interaction of MenSCs with an inflammatory environment could enhance their beneficial properties. Future investigations should be directed toward exploring the specific mechanisms involved in the enhanced effect of MenSCs on wound healing.

## Author Contributions

AL-G, MD, VC, PG, MKG, CS, FE, ME, FA-M, and JC performed the experiments. JB performed the histological analysis. JC and MK conceived, designed, and analyzed the data. JC, CS, and MK wrote the manuscript text. All authors reviewed the manuscript.

## Conflict of Interest Statement

MK is the chief science officer of Cells for Cells and Consorcio Regenero. JC and FA-M received stipends from Cells for Cells. The other authors declare that the research was conducted in the absence of any commercial or financial relationships that could be construed as a potential conflict of interest.

## Footnotes

1 http://www.isip.uni-luebeck.de/index.php?id=150&L=2%255

2 www.clinicaltrials.gov/
