# Supplementary material for: The Reparative Abilities of Menstrual Stem Cells Modulate the Wound Matrix Signals and Improve Cutaneous Regeneration
Source: Front Physiol. 2018 May 14;9:464. doi: 10.3389/fphys.2018.00464 (PMC5960687; doi:10.3389/fphys.2018.00464)
Supplement: Supplementary file 1 [file Table_1.DOCX]

**Supplemental Table 1**. Primers Sequences used to quantify mRNA expression levels in experiments *in vitro* and *in vivo.*

| **Gen** | **Sequence** | |
| --- | --- | --- |
| **Human** | **Forward** | **Reverse** |
| *hGapdh* | F 5ʹ-GGTCTCCTCTGACTTGAACA-3ʹ; | R 5ʹ-GTGAGGGTCTCTCTCTTCCT-3ʹ |
| *hMmp1* | F 5’-GGGAGATCATCGGGACAACTC-3’ | R 5’-GGGCCTGGTTGAAAAGCAT-3’ |
| *hMmp3* | F 5’-CTGGACTCCGACACTCTGGA-3’ | R 5’-CAGGAAAGGTTCTGAAGTGACC-3’ |
| *hMmp10* | F 5’-TGCTCTGCCTATCCTCTGAGT-3’ | R 5’-TCACATCCTTTTCGAGGTTGTAG-3’ |
| *hTimp1* | F 5’-GATACTTCCACAGGTCCCACAAC-3’ | R 5’-GCAAGAGTCCATCCTGCAGTT-3’ |
| *hTimp2* | F 5’-CGACATTTATGGCAACCCTATCA-3’ | R 5’- CAGGCCCTTTGAACATCTTTATCT-3’ |
| *hTimp3* | F 5’-AACTTGGGTGAAGGCTGAGTGT-3’ | R 5’-CCTCACCAAGGCCTAACAGATG-3’ |
| *hIl-1b* | F 5’-CTGAGCTCGCCAGTGAAA T-3’ | R 5’-AGGAGCACTTCATCTGTTTAGG-3’ |
| *hIL-6* | F 5’-AAATTCGGTACATCCTCGACGGCA-3’ | R 5’-AGTGCCTCTTTGCTGCTTTCACAC-3’ |
| *hIL-8* | F 5’- GAATGGGTTTGCTAGAATGTGATA-3’ | R 5’- CAGACTAGGGTTGCCAGATTTAAC-3’ |
| *hbFgf* | F 5’-AGA AGA GCG ACC CTC ACA TCA-3’ | R 5’-CGG TTA GCA CAC ACT CCT TTG-3´ |
| *hHgf* | F 5’-CACCACACCGGCACAAATTC-3’ | R 5’-ATTGCACAGTACTCCCAGCG-3’ |
| *hGm-csf* | F 5’-CCTGAGTAGAGACACTGCTGC-3’ | R 5’-CCTGCTTGTACAGCTCCAG-3’ |
| *hTsg6* | F 5’-TCATGTCTGTGCTGCTGGATG-3’ | R 5 -GGGCCCTGGCTTCACAA-3’ |
| *hSerpine-2* | F 5’-TGCTGGTGAATGCCCTCTACT-3’ | R 5’-CGGTCATTCCCAGGTTCTCTA-3’ |
| *hFn1* | F 5’-GGTGACACTTATGAGCGTCCTAAA-3’ | R 5’-AACATGTAACCACCAGTCTCATGTG-3’ |
| *hPdgfa* | F 5’-TAG GGA GTG AGG ATT CTT TGG-3’ | R 5’-CAC TCC AAA TGC TCC TCT AAC-3’ |
| *hPdgfb* | F 5’-TTGGACCTGAACATGACCCG-3’ | R 5’-TCAGCAATGGTCAGGGAACC-3’ |
| *hVegf* | F 5’-ACACATTGTTGGAAGAAGCACCC-3’ | R 5’-AGGAAGGTCAACCACTCACACACA-3’ |
| *hCol1a2* | F 5’-AAGGTCATGCTGGTCTTGCT-3’ | R 5’-GACCCTGTTCACCTTTTCCA-3’ |
| *hCol3a1* | F 5’-GGGAACAACTTGATGGTGCTACT-3’ | R 5’-TCAGACATGAGACTCTTTGTGCAA-3’ |
| *hEln* | F 5’-GGCCATTCCTGGTGGAGTTCC-3’ | R 5’-AACTGGCTTAAGAGGTTTGCCTCCA-3′ |
| *hTgfb1* | F 5’-ACAATTCCTGGCGATACCTCAGCA-3’ | R 5’-TGCAGTGTGTTATCCCTGCTGTCA-3’ |
| *hTgfb2* | F 5’-AGAGTGCCTGAACAACGGATT-3’ | R 5’-CCATTCGCCTTCTGCTCTT-3′ |
| *hTgfb3* | F 5’-TGGACTTCGGCCACATCAAGAAGA-3’ | R 5’-TGTTGTAAAGGGCCAGGACCTGAT-3’ |
| *hAng1* | F 5’-GGACAGCAGGAAAACAGAGC-3’ | R 5’-CACAAGCATCAAACCACCAT-3’ |
| *hCcl7* | F 5’-GCTCAGCCAGTTGGGATTA-3’ | R 5’-CAGCCTCTGCTTAGGGATTT-3’ |
| *hCxcl12* | F 5’-TGCCCTTCAGATTGTAGCC-3’ | R 5’-CAGGTACTCCTGAATCCACTTT-3’ |
| *hSod1* | F 5’-CATCAGCCCTAATCCATCTGA-3’ | R 5’-CGCGACTAACAATCAAAGTGA-3’ |
| **Mouse** | **Forward** | **Reverse** |
| *mbactin* | F 5’-GATTACTGCTCTGGCTCCTA-3’ | R 5’-ATCGTACTCCTGCTTGCTGA-3’ |
| *mCol1* | F 5’-GCTCCTCTTAGGGGCCACT-3’ | R 5’-CCACGTCTCACCATTGGGG-3’ |
| *mCol-3a1* | F 5’-CTGTAACATGGAAACTGGGGAAA-3’ | R 5’-CCATAGCTGAACTGAAAACCACC-3’ |
| *mSerpine2* | F 5’-TTCAGCCCTTGCTTGCCTC-3’ | R 5’-ACACTTTTACTCCGAAGTCGGT-3’ |
| *mSod2* | F 5’-CAGACCTGCCTTACGACTATGG-3’ | R 5’-CTCGGTGGCGTTGAGATTGTT-3’ |
| *mIcamp1* | F 5’-GTGATGCTCAGGTATCCATCCA-3’ | R 5’-CACAGTTCTCAAAGCACAGCG-3’ |
| *miNOS* | F 5’-AGTTCTGCGCCTTTGCTCAT-3’ | R 5’-AGTGAAGCGTTTCGGGATCT-3’ |
| *mIl-6* | F 5’- CCTTCCTACCCCAATTTCCA -3’ | R 5’- GGCATAACGCACTAGGTTTG -3’ |
| *mIL-8* | F 5’- GGCTACTGTTGGCCCAATTA-3’ | R 5’- GAGGTCTCCCGAATTGGAAA-3’ |
| *mTgfb1* | F 5’-AGTGTGGAGCAACATGTGGA-3’ | R 5’-CAGTGAGCGCTGAATCGAAA-3’ |
| *mTnfa* | F 5’- TTATGGCTCAGGGTCCAACT-3’ | R 5’- GCAGAACTCAGGAATGGACA-3’ |
| *mPlau* | F 5’-GCGCCTTGGTGGTGAAAAAC-3’ | R 5’-TTGTAGGACACGCATACACCT-3’ |
| *mMmp1* | F 5’-AACTACATTTAGGGGAGAGGTGT-3’ | R 5’-GCAGCGTCAAGTTTAACTGGAA-3’ |
| *mMmp2* | F 5’-CAAGTTCCCCGGCGATGTC-3’ | R 5’-TTCTGGTCAAGGTCACCTGTC-3’ |
| *mMmp9* | F 5’-AATCTCTTCTAGAGACTGGGAAGGAG-3’ | R 5’-AGCTGATTGACTAAAGTAGCTGGA-3’ |
| *mVegf* | F 5’-CTGCCGTCCGATTGAGACC-3’ | R 5’-CCCCTCCTTGTACCACTGTC-3’ |
| *mEln* | F 5’-TTGCTGATCCTCTTGCTCAAC-3’ | R 5’-GCCCCTGGATAATAGACTCCAC-3’ |
