# Supplementary material for: The Reparative Abilities of Menstrual Stem Cells Modulate the Wound Matrix Signals and Improve Cutaneous Regeneration
Source: Front Physiol. 2018 May 14;9:464. doi: 10.3389/fphys.2018.00464 (PMC5960687; doi:10.3389/fphys.2018.00464)
Supplement: Supplementary file 2 [file Table_2.DOCX]

**Supplemental Table 2**. Data of the analysed genes in basal and stimulated MSCs with pro-inflammatory cytokines, IL1β and TNFα, for 24 hours. The data are presented as the means ± SEM from three independant experiments. P-value in italics and bold shows significant difference (p≤0.05).

| **Group** | **IL1β+TNFα MenSCs vs MenSCs** | | **IL1β+TNFα UC-MSCs vs UC-MSCs** | | **MenSCs vs UC-MSCs** | | **IL1β+TNFα MenSCs vs IL1β+TNFα UC-MSCs** | |
| --- | --- | --- | --- | --- | --- | --- | --- | --- |
| Gen | Fold induction | P-value | Fold induction | P-value | Fold induction | P-value | Fold induction | P-value |
| *ANGPT1* | 0.698±0.442 | 0.730 | 0.002±0.328 | 0.340 | 1.517±0.357 | ***0.015*** | 2.493±0.558 | ***0.014*** |
| *bFGF* | 4.333±1.556 | 0.114 | 1.463±0.396 | 0.297 | -0.081±0.300 | 0.436 | 1.993±0.716 | 0.297 |
| *CCL7* | 2.488±0.790 | 0.114 | 2.905±0.721 | 0.297 | -0.204±0.062 | ***0.006*** | -0.184±0.051 | ***0.001*** |
| *COL1A2* | 0.499±0.379 | 0.667 | -0.599±0.046 | ***0.036*** | 0.297±0.409 | 0.730 | 1.755±0.431 | ***0.002*** |
| *COL3A1* | 0.034±0.303 | 0.258 | -0.104±0.292 | 0.258 | 0.335±0.378 | 0.931 | 0.630±0.362 | 0.796 |
| *CXCL12* | 0.157±0.295 | 0.252 | 0.294±0.294 | 0.436 | 10.009±2.147 | ***0.001*** | 10.900±2.440 | ***<0.0001*** |
| *CXCL8* | 13.168±3.051 | ***0.000*** | 5.352±0.807 | ***0.000*** | 1.607±0.474 | 0.057 | 4.850±1.124 | ***0.002*** |
| *ELN* | 36.142±9.578 | ***<0.0001*** | 1.388±0.612 | 0.136 | 13.359±2.682 | ***<0.0001*** | 261.231±69.230 | ***<0.0001*** |
| *FN1* | 0.841±0.417 | 0.190 | -0.087±0.327 | 0.297 | -0.265±0.068 | ***0.000*** | -0.456±0.072 | 0.063 |
| *GM-CSF* | 29.096±11.516 | ***<0.0001*** | 8.400±2.373 | ***0.000*** | 5.234±1.139 | ***0.001*** | 18.132±7.177 | ***0.001*** |
| *HGF* | -0.435±0.047 | 0.059 | -0.007±0.261 | 0.136 | -0.212±0.042 | ***0.004*** | -0.158±0.017 | ***0.000*** |
| *IL1B* | 8.222±2.815 | ***0.006*** | 2.415±0.446 | ***0.003*** | -0.088±0.021 | ***<0.0001*** | -0.309±0.100 | ***0.011*** |
| *IL6* | 18.062±4.782 | ***0.000*** | 4.486±1.254 | ***0.001*** | -0.139±0.300 | 1.000 | 2.848±0.796 | ***0.032*** |
| *LCN2* | 3.771±0.629 | ***0.000*** | 4.198±0.914 | ***0.000*** | 5.352±0.223 | ***0.000*** | 4.807±0.802 | ***0.000*** |
| *MMP1* | 5.032±1.059 | ***0.001*** | 0.634±0.459 | 0.222 | 1.099±0.357 | 0.546 | 4.823±1.015 | ***0.001*** |
| *MMP10* | 3.343±0.739 | ***0.003*** | -0.386±0.222 | 0.773 | 9.171±2.206 | ***0.001*** | 50.598±8.700 | ***<0.0001*** |
| *MMP3* | 15.450±2.821 | ***<0.0001*** | 0.582±0.476 | 0.370 | 21.593±3.323 | ***<0.0001*** | 274.049±50.045 | ***<0.0001*** |
| *NFKB1* | 0.910±0.372 | 0.124 | 0.263±0.420 | 0.124 | 1.904±0.181 | ***0.006*** | 2.391±0.338 | ***0.006*** |
| *PDGFA* | -0.130±0.259 | 0.064 | 0.856±0.457 | 1.000 | 1.827±0.363 | ***0.010*** | 0.358±0.395 | 0.796 |
| *PDGFB* | -0.450±0.049 | ***0.005*** | -0.102±0.363 | 0.743 | 791.031±106.988 | ***0.000*** | 522.968±56.599 | ***0.000*** |
| *SERPINE2* | 5.803±0.877 | ***0.000*** | 1.188±0.469 | 0.114 | -0.192±0.052 | ***0.000*** | -0.205±0.265 | 0.370 |
| *SOD1* | -0.544±0.008 | ***0.000*** | -0.702±0.067 | ***0.000*** | 2.413±0.029 | ***0.000*** | 1.868±0.027 | ***0.002*** |
| *TGFB1* | -0.626±0.077 | 0.063 | -0.774±0.053 | 0.289 | -0.256±0.302 | 0.340 | -0.670±0.083 | ***0.019*** |
| *TGFB2* | 2.210±0.535 | ***0.024*** | 0.269±0.368 | 0.860 | -0.472±0.076 | ***0.008*** | 0.682±0.377 | 0.863 |
| *TGFB3* | -0.227±0.045 | ***0.000*** | -0.264±0.037 | ***0.004*** | -0.480±0.081 | 0.059 | -0.413±0.082 | ***0.003*** |
| *TIMP1* | 0.176±0.410 | 0.796 | -0.094±0.331 | 0.436 | -0,168±0.182 | ***0.011*** | -0.207±0.189 | ***0.019*** |
| *TIMP2* | -0.167±0.331 | 1.000 | -0.711±0.025 | ***0.027*** | 2.529±0.262 | ***0.000*** | 3.328±0.209 | ***0.000*** |
| *TIMP3* | -0.428±0.108 | ***0.019*** | -0.069±0.269 | 0.085 | -0.148±0.024 | ***0.000*** | -0.098±0.025 | ***0.000*** |
| *TSG6* | 5.696±1.820 | ***0.003*** | 3.607±0.398 | ***0.000*** | 1.824±0.362 | ***0.047*** | 2.827±1.014 | 0.059 |
| *VEGFA* | 0.546±0.450 | 0.436 | 1.012±0.360 | 0.094 | 0.131±0.347 | 1.000 | -0.211±0.325 | 0.233 |
