# Supplementary figures and images for: The Reparative Abilities of Menstrual Stem Cells Modulate the Wound Matrix Signals and Improve Cutaneous Regeneration
Source: Front Physiol. 2018 May 14;9:464. doi: 10.3389/fphys.2018.00464 (PMC5960687; doi:10.3389/fphys.2018.00464)

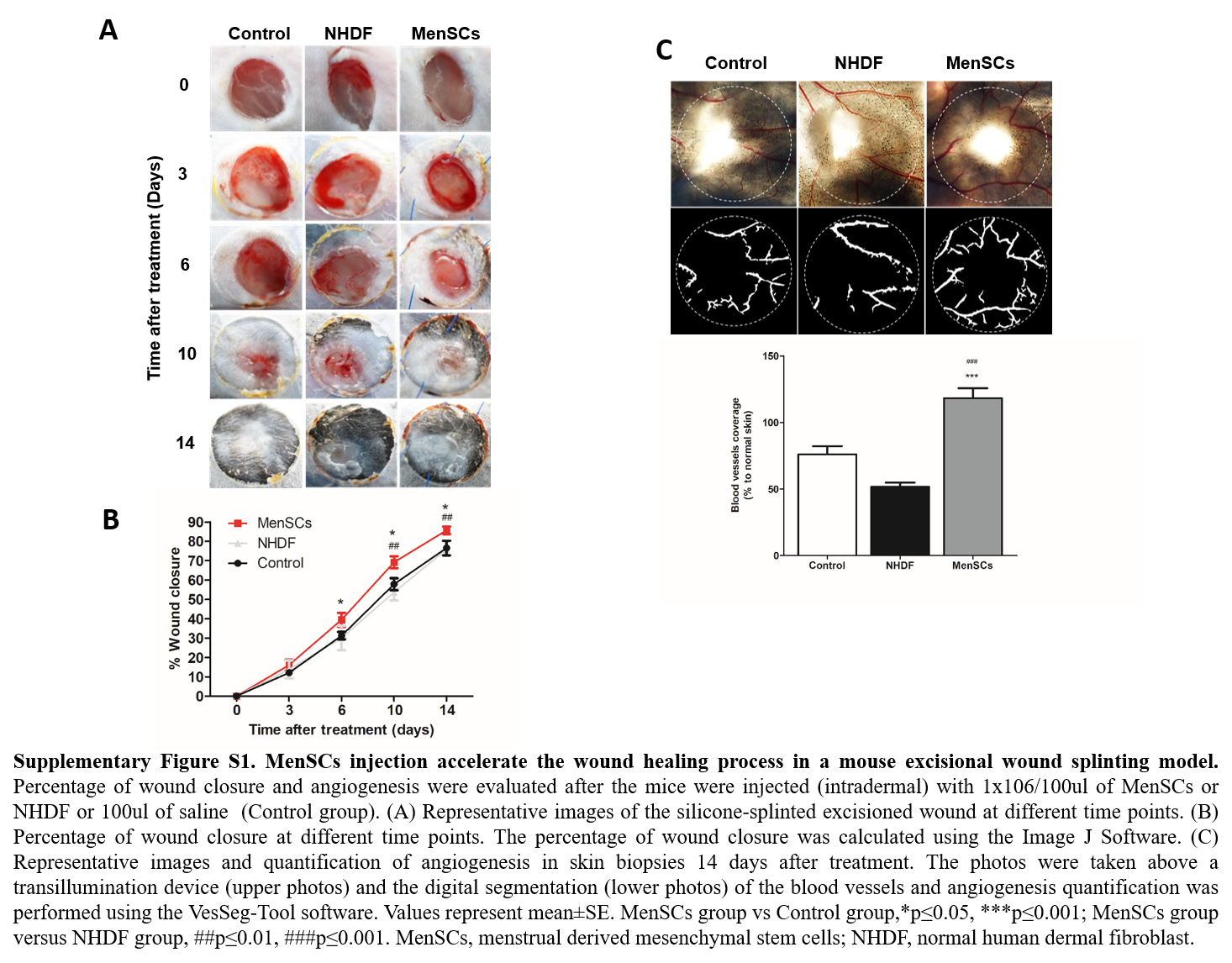

Supplement: Supplementary file 3 [file Image_1.tif]
